# Clinical Performance of Prediction Rules and Nasogastric Lavage for the Evaluation of Upper Gastrointestinal Bleeding: A Retrospective Observational Study

**DOI:** 10.1155/2017/3171697

**Published:** 2017-01-26

**Authors:** Hassan K. Dakik, F. Douglas Srygley, Shih-Ting Chiu, Shein-Chung Chow, Deborah A. Fisher

**Affiliations:** ^1^Duke Gastroenterology Division, Duke University Hospital, Durham, NC, USA; ^2^Austin Gastroenterology, Austin, TX, USA; ^3^Duke Biostatistics Division, Duke University Hospital, Durham, NC, USA; ^4^Durham VA Health Services Research and Development Center, Durham, NC, USA

## Abstract

*Introduction*. The majority of patients with acute upper gastrointestinal bleeding (UGIB) are admitted for urgent endoscopy as it can be difficult to determine who can be safely managed as an outpatient. Our objective was to compare four clinical prediction scoring systems: Glasgow Blatchford Score (GBS) and Clinical Rockall, Adamopoulos, and Tammaro scores in a sample of patients presenting to the emergency department of a large US academic center.* Methods*. We performed a retrospective cohort study of patients during 2008–2010. Our outcome was significant UGIB defined as high-risk stigmata on endoscopy, or receipt of blood transfusion or surgery, or death.* Results*. A total of 393 patients met inclusion criteria. The GBS was the most sensitive for detecting significant UGIB at 98.30% and had the highest negative predictive value (90.00%). Adding nasogastric lavage data to the GBS increased the sensitivity to 99.57%.* Conclusions*. Of all four scoring systems compared, the GBS demonstrated the highest sensitivity and negative predictive value for identifying a patient with a significant UGIB. Therefore, patients with a 0 score can be safely managed as an outpatient. Our results also suggest that performing a nasogastric lavage adds little to the diagnosis UGIB.

## 1. Introduction

Gastrointestinal bleeding (GIB) is a common and serious condition with an estimated 545,000 hospitalizations yearly in the United States (US) [1]. Hospitalizations for upper GIB (UGIB) have a mean length of stay of 2.7–4.4 days and a mean cost of $3,400–$13,000 [2, 3]. Currently, the majority of patients presenting with acute UGIB are admitted for an urgent esophagogastroduodenoscopy (EGD) as it can be difficult to differentiate between those patients at high risk for significant bleeding and those who can be safely managed as an outpatient [4]. Several different prognostic scoring algorithms have been proposed to facilitate clinical decision-making, specifically the determination of hospital admission versus outpatient management. These scoring systems use different combinations of clinical factors and have been evaluated to predict bleeding severity and identify those patients safe for discharge.

In initial studies, these clinical prediction rules have suggested that as many as a third of all patients presenting with an acute UGIB may be safely discharged from the emergency department (ED) to nonurgent follow-up [4]. The best studied scoring systems are the Glasgow Blatchford Score (GBS) and the Clinical Rockall score [4–9]. However, there are minimal data validating these two scoring algorithms in US populations. This may be a barrier to wider use of these scoring systems by ED providers because practices in Europe and Britain, where these scores were developed and predominantly validated, differ from US practices. In particular, the criteria for admission are notably different. Thus, the applicability of these scores in a US health system is unclear. In addition to the GBS and Clinical Rockall scores, the Adamopoulos and Tammaro scores have been developed but only validated in a single publication each [10, 11]. To date, there have been no direct comparison studies to evaluate these four scoring systems in the same patient population to determine their head-to-head performance.

An additional issue in the management of acute UGIB is whether nasogastric (NG) lavage is needed or helpful. NG lavage is a method of attempting to obtain fluid from the stomach and, potentially, the small bowel to determine the location of a possible GIB and help determine the severity of the bleed [12]. The value of NG lavage remains controversial with conflicting results amongst studies [13–15]. Nonetheless, the practice is still common in US institutions.

Our primary goal was to determine which of the scoring systems was best able to discern patients at low risk for significant hemorrhage who could be safely managed as an outpatient. Therefore, we estimated and compared the performance characteristics of GBS, Clinical Rockall, Adamopoulos, and Tammaro scores in identifying a significant UGIB in a sample of US patients who presented to the ED with signs/symptoms consistent with an acute UGIB. Our secondary aim was to evaluate any additional value of NG lavage data when integrated into the most accurate scoring systems.

## 2. Methods

We performed a retrospective cohort study of patients who presented to the Duke University Hospital ED with a diagnosis of UGIB during 2008–2010. The patients were initially identified based on International Classification of Diseases Version 9 (ICD 9) codes for upper GI bleeding (e.g., upper GI bleed, hematemesis, and melena) via the Duke Enterprise Data Unified Content Explorer (DEDUCE) database which is a web-based tool allowing specific clinical data to be extracted by researchers from the electronic medical record [16]. These ICD-9 codes are listed in the following. 

*ICD-9 Codes for GI Bleeding*

456.00 - ESOPHAGEAL VARICES WITH BLEEDING
456.20 - ESOPHAGEAL VARICES IN DISEASES CLASSIFIED ELSEWHERE WITH BLEEDING
456.80 - VARICES OF OTHER SITES
530.20 - ULCER OF ESOPHAGUS WITHOUT BLEEDING
530.21 - ULCER OF ESOPHAGUS WITH BLEEDING
530.70 - GASTROESOPHAGEAL LACERATION
531.10 - ACUTE GASTRIC ULCER WITH PERFORATION WITHOUT OBSTRUCTION
531.20 - ACUTE GASTRIC ULCER WITH HEMORRHAGE AND PERFORATION WITHOUT OBSTRUCTION
531.40 - CHRONIC OR UNSPECIFIED GASTRIC ULCER WITH HEMORRHAGE WITHOUT OBSTRUCTION
531.60 - CHRONIC OR UNSPECIFIED GASTRIC ULCER WITH HEMORRHAGE AND PERFORATION WITHOUT OBSTRUCTION
532.00 - ACUTE DUODENAL ULCER WITH HEMORRHAGE WITHOUT OBSTRUCTION
532.20 - ACUTE DUODENAL ULCER WITH HEMORRHAGE AND PERFORATION WITHOUT OBSTRUCTION
532.40 - CHRONIC OR UNSPECIFIED DUODENAL ULCER WITH HEMORRHAGE WITHOUT OBSTRUCTION
532.60 - CHRONIC OR UNSPECIFIED DUODENAL ULCER WITH HEMORRHAGE AND PERFORATION WITHOUT OBSTRUCTION
533.00 - ACUTE PEPTIC ULCER OF UNSPECIFIED SITE WITH HEMORRHAGE WITHOUT OBSTRUCTION
533.4 - CHRONIC OR UNSPECIFIED PEPTIC ULCER OF UNSPECIFIED SITE WITH HEMORRHAGE WITHOUT OBSTRUCTION
533.60 - CHRONIC OR UNSPECIFIED PEPTIC ULCER OF UNSPECIFIED SITE WITH HEMORRHAGE AND PERFORATION WITHOUT OBSTRUCTION
537.83 - ANGIODYSPLASIA OF STOMACH AND DUODENUM WITH HEMORRHAGE
537.84 - DIEULAFOY LESION (HEMORRHAGIC) OF STOMACH AND DUODENUM
578.00 - HEMATEMESIS
578.10 - BLOOD IN STOOL
578.90 - HEMORRHAGE OF GASTROINTESTINAL TRACT UNSPECIFIED

Next, the electronic medical records (EMR) for each patient were reviewed for inclusion and exclusion criteria. The EMR of eligible subjects was further abstracted using a standard protocol and data collection tool to obtain the data for each scoring algorithm (Table 1).

Inclusion criteria were all patients who presented to the ED with a primary complaint of UGIB as evidenced by hematemesis, melena, coffee ground emesis, or bright red blood per rectum in the appropriate setting (i.e., a brisk GIB as evidenced by hemodynamic changes and hemoglobin drop), from 1/1/2008 through 12/31/2010. Patients must have been seen by a physician in the ED and evaluated with a tentative diagnosis of UGIB.

Patients were excluded if there was no complaint of active bleeding (i.e., only anemia or findings consistent with occult bleeding). In addition, they were excluded if they met any of the following criteria: age <18 years old, found to have lower gastrointestinal or anal bleeding, inpatient status at the time of the onset of bleeding, or lack of sufficient information documented in the EMR to complete the scores.

The clinical factors which were abstracted included age, gender, race/ethnicity, blood urea nitrogen, hemoglobin, international normalized ratio (INR), systolic blood pressure, pulse, history of melena, history of syncope, presence of hepatic disease, history of congestive heart failure, presence of comorbidities (as described in Clinical Rockall score [17]), white blood cell count, general condition (as described by Tammaro score [10]), nasogastric lavage results (categorized as not performed, clear, bilious, coffee grounds, or red blood), use of anticoagulants, and Charleston Comorbidity Index.

The four scores were calculated as described in the literature using the above data (Table 2) [5, 10, 11, 17].

The primary clinical outcome was “significant UGIB” as defined by any of the following: the presence of high-risk stigmata on endoscopy (as defined by Forest classification [18]), receipt of blood transfusion, performance of surgery, or death while in the hospital or, if not admitted, within 30 days. If the patient was not admitted then vital status (dead, alive) or any of the above events at another hospital was investigated by examining outpatient records including clinic visits and outpatient endoscopy. For patients seen in the ED which had no subsequent records in the Duke system, we had IRB permission to contact via letter and then telephone to evaluate vital status and occurrence of any of the other events in the combined outcome.

The primary analysis was calculation of the sensitivity, specificity, positive predictive value (PPV), negative predictive value (NPV), and likelihood ratios of each scoring system to detect a significant UGIB as described above. For each scoring system we used the cutoff value described in the respective original publications.

The GBS score utilizes a combination of laboratory values and clinical parameters to calculate a cumulative numeric score. Any value greater than 0 was considered high risk as described in the original publication and subsequent validation studies [5].

The Rockall score uses similar parameters to arrive at a total numeric score. The initial manuscript described a low rebleeding and mortality rate for cumulative scores 0–2. Scores 3 or greater were noted to have higher incidence of rebleeding as well as a 5-fold increase in mortality [17]. However, this score requires the inclusion of the findings on endoscopy and associated stigmata of bleeding in the setting of peptic ulcer disease. The clinical (or preendoscopy) Rockall score utilizes only the clinical parameters available prior to endoscopic assessment and can be used as a preendoscopy predictor of bleeding risk. This score cutoff is similar to the Blatchford of ≥ 1 being considered high risk.

The Adamopoulos score utilized a simpler formula to calculate risk, which included NG lavage data. The equation was as follows:

(1) Total  score=6  if  fresh  blood  in  NGT+4  if  hemodynamically  unstable+4  if  hemoglobin  less  than  8 g/dL+3  if  WBC  greater  than  12,000/microL.

In their original cohort the authors utilized value of <7 to be consistent with low risk and greater than or equal to 11 was considered high risk. This yielded a sensitivity of 96%, specificity of 98%, and a positive predictive value of 96%. Given that our goal was to favor increased sensitivity we considered any score >7 to be consistent with a significant upper GI bleed [11].

Lastly, the Tamarro score focused on clinical predictors of significant hemorrhage such as vital signs and initial hemoglobin levels to form a basic calculation [10]. In this scoring system a higher numerical score correlated to a lower risk of hemorrhage. The authors divided their sample into three categories representing a low, intermediate, and high risk of rebleeding. However, again, as we were focusing on maximal sensitivity we combined the last 2 categories, namely, the intermediate (score 7–9) and high risk (score ≤6) into one group and considered any score < 10 to be consistent with significant GIB in our analysis.

## 3. Results

A total of 393 patients met our inclusion criteria for upper GIB. Characteristics of the sample are in Table 3; 247 patients were male and 146 were female with a mean age of 54.9 years.

A total of 235 patients met the combined outcome of significant UGIB by either requiring blood products, having high-risk stigmata on endoscopy, needing surgery, or death. The majority of patients had follow-up available in the EMR but 16 patients were contacted because they were discharged from the ED and had no follow-up documentation available.

The results of the statistical analysis are summarized in Table 4. The GBS was the most sensitive for detecting significant UGIB at 98.30%. The specificity was expectedly lower at 22.78%. The NPV was 90.00% and the negative LR was 0.07.

Using a cutoff score of ≥ 1 as described above, the Clinical Rockall score demonstrated a high sensitivity of 92.77% with a specificity of 32.28%. The NPV was 75.00% with a negative LR of 0.22.

The Adamopoulos scoring system had the lowest sensitivity at 29.79% with a higher specificity of 92.41%. The NPV was 46.95% and the negative LR was 0.76. Modifying the cutoffs to a value ≥4 (favoring improved sensitivity) the sensitivity increased to 71.06% with a lesser reduction in specificity to 79.11%, a NPV of 64.77%, and negative LR of 0.37.

Lastly, the Tammaro scoring system had a sensitivity of 79.57%, specificity of 60.76%, and NPV of 66.67%. The negative LR was 0.34.

Adding NG lavage data to the GBS with coffee grounds and red blood serving as a positive result with a value of 1 additional point, the sensitivity increased to 99.57% and the specificity remained unchanged at 22.78%. The NPV increased to 97.3% and the negative LR was 0.02.

The majority of patients in our study were admitted to the hospital for management of UGIB. 82 patients (20.87%) were discharged and managed as outpatients. Of these only 4 (1%) met our criteria for having a significant UGIB and 29 (7.4%) of these patients had a Blatchford score of 0.

## 4. Discussion

One of the most important steps in the management of acute upper GI bleeding occurs in the initial triage of these patients. The clinical presentation of UGIB can vary widely and determining which patients will require urgent evaluation can be difficult. Moreover, this decision is often made by ED providers without the aid of an experienced gastroenterologist. In an effort to avoid discharging patients home with the potential for worsening bleeding, the majority of patients presenting with UGIB are admitted to the hospital for an urgent evaluation, which often culminates in upper endoscopy [4]. On the other hand, if hospital admission is not needed because outpatient evaluation and follow-up are safe, then this cost and patient inconvenience can be avoided.

Several scoring systems have been developed as clinical decision tools prior to endoscopy which may facilitate appropriate triage in the ED. Of these, the best studied is the GBS system which has been validated in numerous studies [4–9, 19, 20]. However, these scorings systems have, for the most part, been evaluated in a non-US patient population. This raises the question of the generalizability of these studies to US practice as the underlying populations and common work-flow patterns could differ. While differences in population characteristics (e.g., age, gender, race/ethnicity, comorbidities) may not affect the sensitivity and specificity of the scoring system, positive and negative predictive values are dependent on prevalence which could vary by patient mix. In addition, we wondered if US providers would reject scoring systems that did not include NG lavage, a frequent triage procedure in the US, and whether the impact of using a score on triage practices (e.g., the proportion of cases admitted that could have been managed out of the hospital) would vary with admission thresholds in different settings.

Our retrospective study is one of the few to test several GIB prediction scoring systems in a US based population. More specifically, we examined the test characteristics on a large sample of patients presenting to the ED of a major academic medical center. It is also the first study to date to directly compare these four scoring algorithms in the same patient population.

Overall, the scoring system with the highest sensitivity was the GBS. In our patient population this scoring system was able to correctly identify patients with evidence of significant upper GI bleeding in nearly 99% of cases. For the purposes of our analysis, we focused on sensitivity and negative predictive values and were less concerned with correspondingly lower specificity.

Compared to the other scoring algorithms, the Clinical Rockall had a high sensitivity but remained inferior to the GBS in all evaluated capacities. The Tammaro score had modest sensitivity with slightly improved specificity compared to the GBS. The NPV of the Clinical Rockall and Tammaro scores were in our opinions inadequate to safely determine patients at low risk of having a significant UGIB who could be discharged without endoscopy. Of note, the original Rockall score included endoscopic data and was developed to guide discharge decisions* after* urgent endoscopy. Therefore, it is not too surprising that the Clinical Rockall score, which does not include any findings from endoscopy, does not perform as well as an initial triage tool prior to endoscopy.

In the largest prior study from the US both the GBS and Clinical Rockall score were evaluated retrospectively with a similar clinical endpoint of significant GI hemorrhage. This analysis revealed sensitivities of 99% and 100% for the GBS and clinical Rockwall, respectively. However, the specificities were only 6% and 4% respectively (using standard cutoff values) [20]. The investigators concluded that the scoring systems were of limited value and recommended instead clinical decision-making in consultation with GI specialists. We agree that careful clinical assessment is important; however, we disagree regarding the value of GBS to aid appropriate triage from the ED. The high sensitivity of the GBS scoring algorithm allows the test to be useful in determining patients appropriate for outpatient management.

A 2015 study suggested that the cutoff for the GBS could be increased to ≤1 to increase the specificity while maintaining a sensitivity of 99% [21]. This change in cutoff would allow more patients to be considered low risk for the purposes of triage. We performed a secondary analysis of the GBS in our data set with a ≤1 cutoff and this resulted in a sensitivity of 96.15%. While we were not able to replicate a sensitivity of 99% our results still suggest that this higher cutoff might be reasonable to consider.

Our study also evaluated the use of NG lavage as a triage tool. This test is still used widely in clinical practice in spite of published test characteristics with sensitivities of 42–84%, specificities of 54–91%, and NPV of 64–85% [15, 16] and data suggesting that use of NG lavage does not significantly alter mortality, hospital length of stay, transfusion requirements, or the need for surgery [22]. Our analysis revealed that incorporation of the NG lavage data into the GBS did improve the sensitivity as well as the NPV with no effect on the specificity. However, the sensitivity increased only marginally and was over 95% prior to addition of the NG lavage data. Therefore, any added benefit from NG lavage appears minimal. While there may be a role for an NG lavage in an effort to clear gastric contents prior to endoscopic evaluation, the value in the diagnostic evaluation of an acute UGIB appears negligible which is consistent with the prior studies.

Although the incidence of UGIB in the US has been decreasing, likely because of wide spread proton pump inhibitor (PPI) therapy, treatment of* Helicobacter pylori (H. pylori)* infection and decreased* H. pylori* incidence [23], the advent of new anticoagulant and antiplatelet agents and increased use of newer cardiac devices and therapies may affect these trends. Regardless, the financial burden of UGIB management on the health care system remains high. Estimated costs for the inpatient management of upper GI bleeding range from $3,180 to $14,301 [24, 25]. Outpatient management of upper GI bleeding has fewer costs, even when endoscopy is required. Use of GBS to facilitate appropriate discharge could decrease costs without compromising patient safety.

Our study was a retrospective analysis and accordingly was subject to certain inherit biases. To avoid ascertainment bias the data were abstracted and verified by multiple researchers and the abstraction protocol was standardized. Moreover, the data points used were largely fixed values and less susceptible to individual interpretation. Patients were called after discharge if outcome data were not complete to avoid attrition bias. This was a single center study at a large academic center and hence may be less clearly representative of other patient populations. However, we do feel that the population was adequately representative of large US academic hospitals and as such the results are generalizable to that clinical setting. Our outcome was significant UGIB which does not have a standard definition clinically or in research. The findings of high-risk stigmata on endoscopy or documentation of intervention (i.e., transfusion, surgery) or death have been used in various combinations in other studies. However, the clinical use of transfusion was not standardized at our institution at the time of the study. While this could have potentially misclassified a patient's outcome, it should not have affected the* relative* performance of the scoring systems. Some of the scoring algorithms were not originally validated for the purposes of triaging significant upper GIB such as the Adamopoulos score which was originally aimed at targeting those in need of urgent endoscopy, or the Clinical Rockall score which aimed to predict risk of death following acute UGIB. For that reason we performed separate analyses using altered cutoff values, which favored increased sensitivity for these scores. Lastly, the analysis of NG lavage was based on clinical practice and may have selected for certain clinical presentations and characteristics of patients. However, our findings did not suggest that this procedure contributed substantially to the diagnosis of a significant UGIB and as such this influence was likely minimal. Moreover, the results obtained are consistent with the majority of recently published studies evaluating the utility of this exam.

In conclusion, of the four scoring systems compared, the GBS demonstrated the highest sensitivity and NPV for identifying a patient with a significant UGIB. Therefore, patients with a 0 score can be safely managed as an outpatient. Our results also suggest that performing an NG lavage adds little to the diagnosis of significant UGIB. In an era of increasing cost consciousness and efforts to reduce unnecessary spending, a simple tool that may facilitate more cost-effective management of UGIB should be considered.

## Figures and Tables

**Table 1:** Clinical parameters used in scoring algorithms.

Scoring algorithms	Objective clinical parameters	Other clinical factors
Blatchford (GBS)	Blood urea nitrogen, hemoglobin level, systolic blood pressure, heart rate	Presentation with melena or syncope Presence of liver disease or heart failure
Clinical Rockall	Age, systolic blood pressure, heart rate	Presence of heart failure, ischemic heart disease, or any major comorbidity
Adamopoulos	Heart rate, systolic blood pressure, hemoglobin level, WBC.	Presence of fresh blood in NGT aspirate
Tamarro	Heart rate, systolic blood pressure, hemoglobin level	General condition (subjective assessment of comorbidities)

**Table:** (a) Tamarro score

Clinical parameter	Score		
	1	2	3
General conditions	Poor	Intermediate	Good
Pulse (beats/min)	>110	90–110	<90
Systolic blood pressure (mmHg)	<90	90–110	>110
Hemoglobin level (g/dL)	≤8	9-10	>10

*T*-score is sum of the corresponding values for each clinical parameters.

≤6 corresponds to *T*1 (high-risk), 7–9 to *T*2 (intermediate-risk), and ≥10 to *T*3 (low-risk).

**Table:** (b) Adamopoulos score

Total points = [6 (if fresh blood in NGT) + 4 (if hemodynamically unstable) + 4 (if hemoglobin < 8 g/dL) + 3 (if WBC > 12,000/*µ*L)].
Score <7 = low risk, score ≥11 = high risk.

**Table:** (c) Clinical Rockall score (before endoscopy)

Clinical parameter	Score			
	0	1	2	3
Age (years)	<60	60–79	≥80	
Shock	“No shock” SBP ≥ 100 mmHg, pulse < 100/min	“Tachycardia” SBP ≥ 100 mmHg, pulse ≥ 100/min	“Hypotension” SBP < 100 mmHg	
Comorbidity	No major comorbidities		CHF, ischemic heart disease, any major comorbidity	Renal failure, liver failure, disseminated malignancy

**Table:** (d) Blatchford score

Admission risk marker	Score
Blood urea (mmol/L)	
≥6.5 <8.0	**2**
≥8.0 <10.0	**3**
≥10.0 ≤25.0	**4**
>25	**6**
Hemoglobin (g/dL) males	
≥12.0 <13.0	**1**
≥10.0 <12.0	**3**
<10.0	**6**
Hemoglobin (g/dL) females	
≥10.0 <12.0	**1**
<10.0	**6**
Systolic blood pressure	
100–109	**1**
90–99	**2**
<90	**3**
Other markers	
Pulse ≥ 100 (per min)	**1**
Presentation with melena	**1**
Presentation with syncope	**2**
Hepatic disease	**2**
Cardiac failure	**2**

**Table 3:** Demographics and endpoints.

		*N*	%
Age (mean)	54.94		
Male		247	**62.85**
Female		146	**37.15**
Race/ethnicity	White	213	**54.2**
	African American	163	**41.48**
	Asian	4	**1.02**
	Hispanic	6	**1.53**
	Native American	7	**1.78**
EGD performed		215	**54.85**
Endoscopic therapy utilized if EGD done		57	**26.51**
Blood transfusion needed		155	**39.44**
Need for surgery		1	**0.25**
Death during hospitalization		28	**7.12**

**Table 4:** Test parameters for each scoring system.

Prediction scoring system	Sensitivity	95% CI	Specificity	95% CI	NPV	95% CI	Negative LR	95% CI
Blatchford (GBS)	**98.30**	96.64–99.95	22.78	16.24–29.33	90.00	80.70–99.30	0.07	0.0–0.15
GBS + NGL	**99.57**	98.74–100.00	22.78	16.24–29.33	97.30	92.07–100.00	0.02	−0.02–0.06
Clinical Rockall Score (≥1)	**92.77**	89.45–96.08	32.28	24.99–39.57	75.00	64.71–85.29	0.22	0.11–0.34
Adamopoulos (≥7)	**29.79**	23.94–35.63	92.41	88.27–96.54	46.95	41.40–52.49	0.76	0.69–0.83
Adamopoulos (≥4)	**71.06**	65.27–76.86	79.11	72.78–85.45	64.77	58.03–71.51	0.37	0.29–0.44
Tamarro	**79.57**	74.42–84.73	60.76	53.15–68.37	66.67	58.97–74.37	0.34	0.24–0.43
